# Room-temperature continuous-wave topological Dirac-vortex microcavity lasers on silicon

**DOI:** 10.1038/s41377-023-01290-4

**Published:** 2023-10-24

**Authors:** Jingwen Ma, Taojie Zhou, Mingchu Tang, Haochuan Li, Zhan Zhang, Xiang Xi, Mickael Martin, Thierry Baron, Huiyun Liu, Zhaoyu Zhang, Siming Chen, Xiankai Sun

**Affiliations:** 1 Department of Electronic Engineering, The Chinese University of Hong Kong, Shatin, New Territories Hong Kong SAR, China; 2 School of Science and Engineering, The Chinese University of Hong Kong, Shenzhen, Guangdong 518172 China; 3 Department of Electronic and Electrical Engineering, University College London, London, WC1E 7JE UK; 4 Université Grenoble Alpes, CNRS, CEA-LETI, MINATEC, Grenoble INP, LTM, F-38054 Grenoble, France

**Keywords:** Semiconductor lasers, Photonic devices

## Abstract

Robust laser sources are a fundamental building block for contemporary information technologies. Originating from condensed-matter physics, the concept of topology has recently entered the realm of optics, offering fundamentally new design principles for lasers with enhanced robustness. In analogy to the well-known Majorana fermions in topological superconductors, Dirac-vortex states have recently been investigated in passive photonic systems and are now considered as a promising candidate for robust lasers. Here, we experimentally realize the topological Dirac-vortex microcavity lasers in InAs/InGaAs quantum-dot materials monolithically grown on a silicon substrate. We observe room-temperature continuous-wave linearly polarized vertical laser emission at a telecom wavelength. We confirm that the wavelength of the Dirac-vortex laser is topologically robust against variations in the cavity size, and its free spectral range defies the universal inverse scaling law with the cavity size. These lasers will play an important role in CMOS-compatible photonic and optoelectronic systems on a chip.

## Abstract (web summary)

The first topological Dirac-vortex microcavity lasers on silicon are experimentally realized. Operating under room-temperature continuous-wave conditions, these lasers mark an important step toward integrating topological lasers with CMOS-compatible optoelectronic systems.

## Graphical abstract

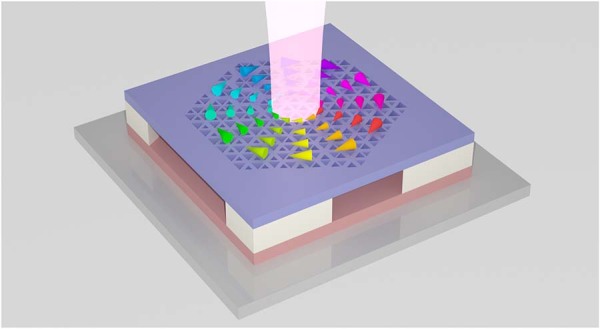

## Introduction

With the explosive growth of data traffic, it is highly desired to develop hybrid photonic integrated circuits (PICs) combining various optical components including lasers, modulators, waveguides, and detectors on a single chip^1^. Silicon is an outstanding material for PICs due to its unique strength in modulating, waveguiding, and detecting photons^2^, but realizing high-performance laser sources in silicon remains challenging^3^. Monolithic integration of III–V quantum-dot (QD) lasers on silicon^4,5^ is considered as a promising strategy to solve this problem because of its lower substrate cost, higher yield, and better CMOS compatibility compared with conventional heterogeneous integration methods^6^. Various III–V QD lasers have been demonstrated on silicon under room-temperature continuous-wave conditions, including distributed-feedback lasers^4^, ridge-waveguide lasers^7^, microring/microdisk lasers^8^, and photonic crystal cavity lasers^9^. Recently, topology as a mathematical concept has attracted intense interests in the realm of optics^10,11^ and is revolutionizing the design strategies for lasers with many surprising properties^12–16^. Topological lasers have been demonstrated using zero-dimensional defect states in Su–Schrieffer–Heeger lattices^12,17,18^ or corner states in higher-order topological insulators^13,19,20^, and one-dimensional edge states in quantum Hall^14,21^, quantum spin Hall^22^, or quantum valley Hall^15,23,24^ topological insulators. However, these topological lasers are not monolithically integrated on silicon substrates and cannot operate under room-temperature continuous-wave conditions, which strongly limit their potential applications in next-generation silicon-based PICs.

Recently, Dirac-vortex state^25^, an analog of the well-known Majorana bound state (MBS) in superconductor electronic systems^26^, has been implemented in the photonic and phononic domains as a new strategy to provide tight and robust confinement of classical waves^25,27,28^. Such Dirac-vortex cavities are mostly based on the Kekulé distortion scheme^29^ and possess a larger free spectral range (FSR) than that of most existing optical cavities, which is an advantage for realizing single-mode surface-emitting lasers. However, previous demonstrations of the Dirac-vortex cavities are mostly limited to passive photonic systems^25,27^, and laser emission from an active Dirac-vortex cavity remains experimentally elusive. Additionally, previous photonic Dirac-vortex cavities based on the conventional Kekulé distortion scheme^29^ unavoidably exhibit vector far-field patterns, which are unsuitable for many applications requiring linearly polarized lasers.

Here, we experimentally demonstrated room-temperature continuous-wave Dirac-vortex topological lasers at a telecom wavelength from InAs/InGaAs QD materials monolithically grown on an on-axis silicon (001) substrate. We designed and fabricated the Dirac-vortex photonic crystal lasers by harnessing an auxiliary orbital degree of freedom in topological insulators that has been recently discovered in a nanomechanical system^30^. By doing so, we could control the near-field of the Dirac-vortex cavities to obtain linearly polarized far-field emission. We observed vertical laser emission from such cavities under continuous-wave optical pumping at room temperature. We compared the experimental far-field patterns with the simulated results and confirmed that the laser emission indeed occurs in the topologically protected MBS. Moreover, we fabricated the Dirac-vortex cavities with various cavity sizes and verified that their lasing wavelengths are always near the Dirac frequency due to the topological protection. Besides, we confirmed that the FSR of the Dirac-vortex lasers is unprecedentedly large and defies the universal inverse scaling law of FSR ∝ *V*^−1^, where *V* is the cavity modal volume. Our Dirac-vortex QD lasers not only are promising light sources for next-generation silicon-based PICs with topological robustness, but also open the door to exploration of various phenomena such as non-Hermiticity^31^, bosonic nonlinearity^32^, and quantum electrodynamics^33^ in the context of topological MBS.

## Results

**Fig. 1:**
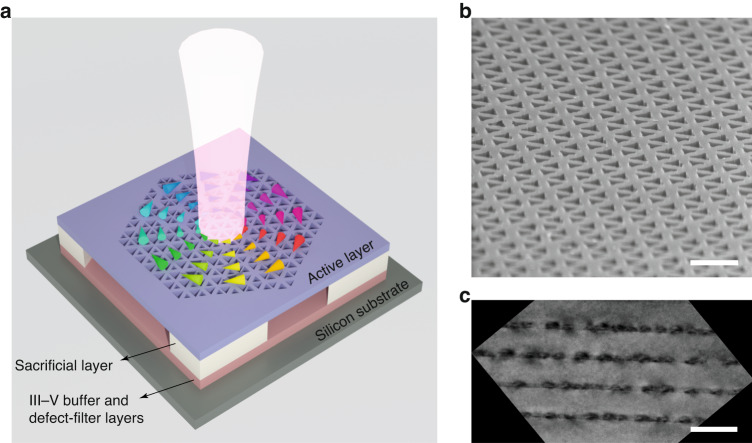
Topological Dirac-vortex microcavity lasers on silicon. **a** Conceptual illustration of a topological Dirac-vortex microcavity laser fabricated on a silicon substrate. The photonic crystal structure was defined in the active layer and suspended by partially removing the sacrificial layer. The III–V buffer and defect-filter layers were carefully optimized to minimize the effects of lattice mismatch between the III–V materials and silicon substrate. **b** Tilted-view scanning electron microscope image of the fabricated topological Dirac-vortex photonic crystal cavity. Scale bar, 500 nm. **c** Cross-sectional bright-field transmission electron microscope image of the active layer containing four-stack InAs/InGaAs QD layers. Scale bar, 100 nm. Adapted with permission from Ref. 34. © 2022 American Chemical Society

Figure 1a is a conceptual illustration of a fabricated Dirac-vortex topological laser based on InAs/InGaAs QDs epitaxially grown on an on-axis silicon (001) substrate. The photonic crystal was defined in the 362-nm-thick active layer and suspended by partially removing the 1-μm-thick Al_0.6_Ga_0.4_As sacrificial layer. The active layer provides tight confinement to light in the vertical direction due to its high refractive index (~3.4). Figure 1b is a tilted-view scanning electron microscope image of the fabricated topological Dirac-vortex photonic crystal cavity. The active layer consists of two symmetric 40-nm-thick Al_0.4_Ga_0.6_As cladding layers and four layers of InAs/In_0.15_Ga_0.85_As dot-in-well structures separated by 50-nm GaAs spacer layers (see Fig. S4 in the Supplementary Information). Figure 1c is a cross-sectional bright-field transmission electron microscope image of the four-stack InAs/InGaAs QD layers^34^. To obtain these high-quality InAs/InGaAs QD layers, the growth process of the III–V buffer and defect-filter layers shown in Fig. 1a was carefully optimized to minimize the effects of lattice mismatch between the III–V materials and silicon substrate (see Fig. S4 in the Supplementary Information).

**Fig. 2:**
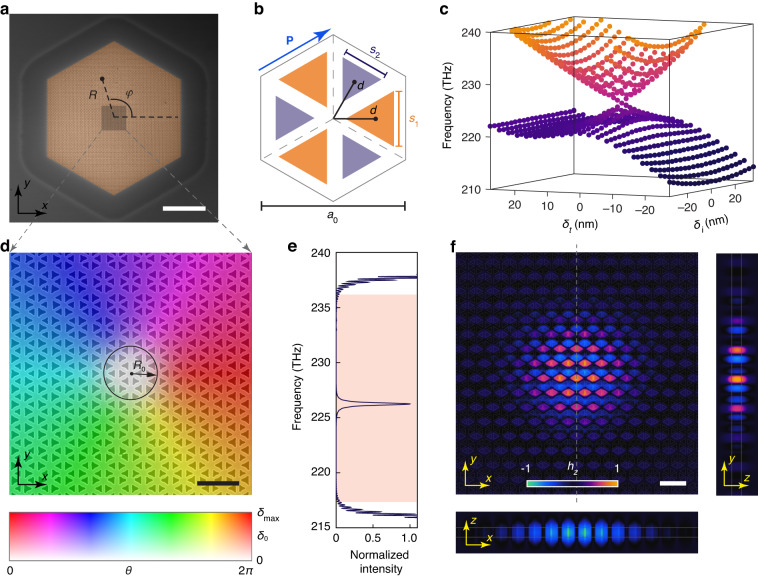
Design and fabrication of the Dirac-vortex laser cavity. **a** Scanning electron microscope image of the fabricated Dirac-vortex topological photonic crystal laser. *R* and *φ* are respectively the radial and angular coordinates of the real space. Scale bar, 10 µm. **b** Illustration of the detailed structure in a unit cell. The lattice constant of the hexagonal photonic crystal is *a*_0_ = 641 nm. Each unit cell contains six triangular holes that can be classified into two groups (colored in purple and orange). The side lengths of the two groups of triangular holes are governed by parameters (*s*_1_, *s*_2_) = (${s}_{0}+{\delta }_{i}$, ${s}_{0}-{\delta }_{i}$) with *s*_0_ = 220 nm. The relative distance from the centers of these triangular holes to the center of the unit cell is *d* = *a*_0_/3 − *δ*_*t*_. Here, ${\delta }_{i}$ breaks the *C*_2_ inversion symmetry and *δ*_*t*_ breaks the *T*_P_ translational symmetry along the vector **P** (marked by the blue arrow). **c** Simulated eigenfrequencies of the bulk states at the Γ point of the first Brillouin zone with different values of *δ*_*i*_ and *δ*_*t*_. **d** Scanning electron microscope image of the photonic crystal structure near the vortex center. It is color-coded by the spatially varying parameters *δ*_0_(*R*) = *δ*_max_·[tanh(*R*/*R*_0_)]^4^ and *θ*(*φ*) = *φ*, where *R*_0_ defines the size of the region with a near-zero value of *δ*_0_(*R*). Scale bar, 1 µm. **e** Simulated normalized intensity spectrum of the Dirac-vortex cavity. A topological Majorana bound state exists in the bulk bandgap (pink region). **f** Simulated modal profiles (*h*_*z*_ component) of the Majorana bound state. Scale bar, 1 µm

Figure 2a is a scanning electron microscope image of the fabricated topological photonic crystal with a hexagonal lattice. Figure 2b shows the detailed structure in a unit cell. The lattice constant of the photonic crystal is *a*_0_ = 641 nm. Each unit cell contains six triangular holes that can be classified into two groups due to the *C*_3_ rotational symmetry. The relative distance from the centers of these triangular holes to the center of the unit cell is *d* = *a*_0_/3 − *δ*_*t*_, where a nonzero *δ*_*t*_ breaks the *T*_P_ translational symmetry of the crystal along the vector **P** (blue arrow in Fig. 2b). The sizes of the two groups of triangular holes are governed by parameters (*s*_1_, *s*_2_) = (${s}_{0}+{\delta }_{i}$, ${s}_{0}-{\delta }_{i}$), where *s*_0_ = 220 nm is the average side length of the holes and *δ*_*i*_ breaks the *C*_2_ inversion symmetry of the crystal. It is interesting to note that photonic crystals with nonzero values of *δ*_*t*_ and *δ*_*i*_ correspond respectively to quantum spin Hall^33^ and quantum valley Hall^15^ photonic topological insulators. The simulated eigenfrequencies of the bulk states at the Γ point of the first Brillouin zone exhibit a doubly degenerate anisotropic cone-like dispersion relation in the parameter space defined by *δ*_*t*_ and *δ*_*i*_ (Fig. 2c). The polar coordinates (*δ*_0_, *θ*) can be defined according to (*δ*_*t*_, *δ*_*i*_) = *δ*_0_(*α*·sin*θ*, cos*θ*), with *α* = 0.65 (0.33) for *δ*_*t*_ > 0 (*δ*_*t*_ < 0) such that the opened bandgap at the Γ point has less *θ* dependence (see Fig. S5 in the Supplementary Information). Here, *θ* represents an auxiliary orbital degree of freedom that can be used to construct the MBS. We theoretically obtained the effective bulk Hamiltonian mathematically identical to the Jackiw–Rossi model^26^: $H({\bf{k}})={v}_{\text{D}}\cdot ({\sigma }_{x}{k}_{x}+{\sigma }_{y}{k}_{y})+\frac{{\triangle }_{0}}{2}{\sigma }_{z}\left({\tau }_{x}\cos \theta -{\tau }_{y}\sin \theta \right)$, where *σ*_*x*_, *σ*_*y*_, *τ*_*x*_, and *τ*_*y*_ are the Pauli matrices, **k** = (*k*_*x*_, *k*_*y*_) is the wave vector, *v*_D_ is the effective Fermi velocity near the Γ point, and Δ_0_ = *ςδ*_0_ is the opened bandgap and is proportional to geometric parameter *δ*_0_ with a proportionality constant *ς*.

Figure 2d shows the detailed structure near the center of the fabricated photonic crystal, which is color-coded by the spatially varying parameters *δ*_0_(*R*) = *δ*_max_·[tanh(*R*/*R*_0_)]^4^ and *θ*(*φ*) = *φ*. *R* and *φ* are the polar coordinates of the real space as marked in Fig. 2a, *R*_0_ defines the size of the MBS, and *δ*_max_ controls the opened bulk bandgap based on Δ_max_ = *ςδ*_max_ at *R* >> *R*_0_. The modal profile (*z* component of magnetic field) of the MBS was theoretically obtained:

1 $${h}_{z}(R,\varphi )={g}_{0}\left(R\right)\cdot \left|{\psi }_{0}\right\rangle$$

which is determined by an envelope function *g*_0_(*R*) controlling the modal volume *V* of the MBS and a periodic Bloch mode $|{\psi }_{0}\rangle$ controlling the detailed modal profile in each unit cell (see Supplementary Information Sec. 1). The device in Fig. 2d adopted the parameters *R*_0_ = *a*_0_ and *δ*_max_ = 35 nm. Through three-dimensional finite-difference time-domain simulation, we found that such a device supports a MBS in the bulk bandgap (Fig. 2e). The simulated modal profile of this topological MBS is mirror-symmetric with respect to the dashed gray line in Fig. 2f, because the device structure has the same type of symmetry. Note that the modal profiles of the MBS in our design are different from those based on the Kekulé distortion scheme. This is because we used the auxiliary degree of freedom recently discovered in a nanomechanical system. Consequently, this different scheme leads to the different choice of the Bloch mode $|{\psi }_{0}\rangle$. Compared with the Kekulé distortion scheme^25^, ours is more suitable for practical lasers because it exhibits linearly polarized emission rather than vector-beam emission. Compared with conventional laser designs such as vertical-cavity surface-emitting lasers (VCSELs) and Fabry–Pérot (FP) lasers, the Dirac-vortex cavity lasers exhibit a fundamentally different scaling relationship between their FSR and modal volume *V*. Actually, the conventional laser designs including VCSELs and FP lasers follow a relationship of FSR ∝ *V*^−1^, while the Dirac-vortex cavity lasers follow a relationship of FSR ∝ *V*^−1/2^ and thus possess a larger FSR for a given *V*. This is because the Dirac-vortex cavity resonance is topologically pinned to the Dirac point, which has zero density of states in contrast to nonzero density of states in the conventional structures. Therefore, the Dirac-vortex lasers can exhibit better single-modedness than conventional lasers, and this advantage becomes more pronounced as the modal volume *V* increases.

**Fig. 3:**
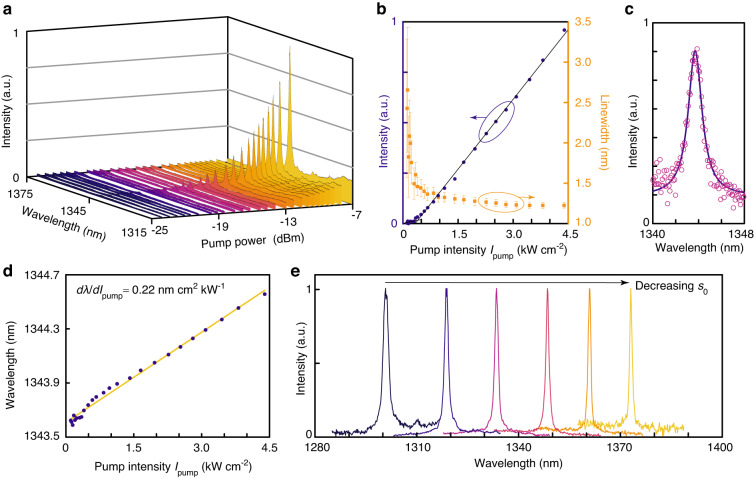
Experimental characterization of the topological Dirac-vortex microcavity lasers. **a** Measured μ-PL spectra of the Dirac-vortex lasers with structural parameters *R*_0_ = 2*a*_0_ and *δ*_max_ = 35 nm as a function of the pump power. **b** Measured μ-PL intensity (purple dots) and linewidth (orange squares) as a function of the pump intensity *I*_pump_. The error bars represent the standard deviation in the linewidth fitting. The lasing threshold is *I*_th_ = 0.4 kW cm^−2^. **c** μ-PL spectrum measured at pump intensity *I*_pump_ = 0.395 kW cm^−2^. The Lorentzian fit (purple solid line) indicates a linewidth of *δλ* = 1.461 nm. **d** Measured lasing wavelength *λ* (purple dots) as a function of the pump intensity *I*_pump_, where a linear fit (orange line) suggests a linear coefficient of *dλ*/*dI*_pump_ = 0.22 nm cm^2^ kW^−1^. **e** Measured normalized lasing spectra from the Dirac-vortex lasers with varying *s*_0_ and fixed *R*_0_ = *a*_0_ and *δ*_max_ = 35 nm. The lasing wavelength can be tuned in a range wider than 70 nm

The microphotoluminescence (μ-PL) measurement was performed using a 632.8-nm continuous-wave pump laser at room temperature. The pump laser was focused onto the center of the Dirac-vortex cavity using an object lens. The light emitted from the topological cavity was collected by the same lens. Figure 3a shows the measured spectra of a topological Dirac-vortex laser with structural parameters *R*_0_ = 2*a*_0_ and *δ*_max_ = 35 nm. Increasing pump power leads to enhanced light emission, with a peak wavelength *λ* at ~1344 nm. The difference between the measured lasing wavelength (*λ* = 1344 nm) and the simulated resonant wavelength (*λ* = 1326 nm) is attributed to a slight deviation of the structural parameter *s*_0_ in device fabrication. Figure 3b shows the measured lasing intensity as a function of the pump intensity *I*_pump_ (purple dots), which indicates a threshold pump intensity *I*_th_ = 0.4 kW cm^−2^. The linewidth-narrowing effect was also observed, which confirms the lasing operation. The lasing linewidth *δλ* fitted from the measured spectra (orange squares) reduces from 2.659 to 1.225 nm as *I*_pump_ increases to 11*I*_th_ (~4.4 kW cm^−2^). Figure 3c shows the measured spectrum (magenta open circles) and the corresponding Lorentzian fit (purple solid line) at *I*_pump_ = 0.395 kW cm^−2^, which indicate a linewidth of *δλ* = 1.461 nm and the corresponding cavity *Q* factor of *λ*/*δλ* = 920. Compared with the simulated cavity *Q* factor of 1590, the experimental *Q* factor is lower, which can be attributed to unavoidable imperfections in device fabrication. We also theoretically analyzed the lasing actions of Dirac-vortex cavity using coupled rate equations. Here, the laser operation exhibits a soft turn-on behavior, suggesting that the spontaneous emission coupling efficiency *β* is high. The carrier density *N* and photon density *P* in the cavity are described by the following rate equation model:

2 $$\left\{\begin{array}{l}\frac{dN}{dt}=\eta \frac{{P}_{in}}{\hslash {\omega }_{p}{V}_{a}}-\frac{N}{{\tau }_{r}}-\frac{N}{{\tau }_{nr}}-{v}_{g}g(N)P\\ \frac{dP}{dt}=\Gamma {v}_{g}g(N)P+\Gamma \beta \frac{N}{{\tau }_{r}}-\frac{P}{{\tau }_{P}}\end{array}\right.$$

where *η* is the absorption ratio of the pump laser in the active region, *ω*_*p*_ is the angular frequency of the pump laser, *V*_*a*_ is the active volume, *τ*_*r*_ (*τ*_*nr*_) is the radiative (nonradiative) recombination lifetime, *v*_*g*_ is the group velocity, and Γ is the confinement factor. A logarithmic gain function *g*(*N*) = *g*_0_log(*N*/*N*_*tr*_) is assumed, where *N*_*tr*_ is the transparency carrier density. By theoretical fitting of the experimental results, we estimated that the spontaneous emission coupling efficiency *β* is ~0.1 (see Fig. S6 in the Supplementary Information). As shown in Fig. 3d, increasing *I*_pump_ leads to redshift in the peak wavelength of the Dirac-vortex laser with a linear coefficient of *dλ*/*dI*_pump_ = 0.22 nm cm^2^ kW^−1^ due to the thermal effect. In addition, we fabricated a series of Dirac-vortex lasers with varying *s*_0_ and fixed *R*_0_ = *a*_0_ and *δ*_max_ = 35 nm. The normalized lasing spectra in Fig. 3e indicate that the resonant wavelength of the topological Dirac-vortex lasers redshifts with a decreased *s*_0_, which can be harnessed for tuning the lasing wavelength in a wide range of >70 nm.

**Fig. 4:**
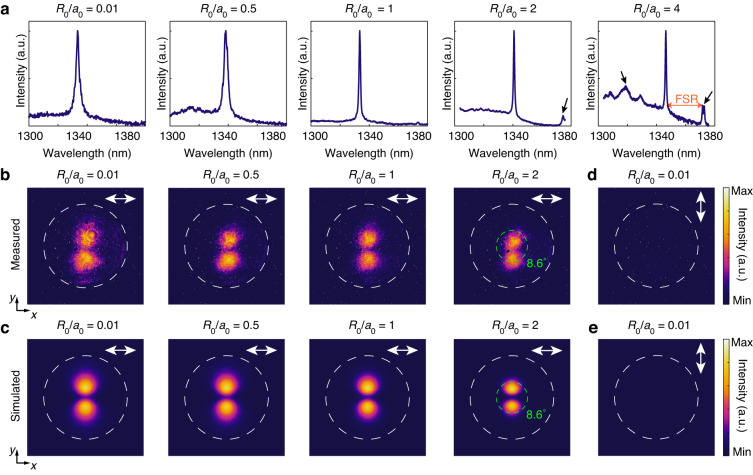
Lasing characteristics of the topological Dirac-vortex microcavity lasers with different cavity sizes. **a** Measured normalized lasing spectra from the Dirac-vortex lasers with *R*_0_/*a*_0_ = 0.01, 0.5, 1, 2, and 4. Some sidebands (marked by the black arrows) emerge in the lasing spectrum of the device with *R*_0_/*a*_0_ = 2 and 4. The FSR is determined by the distance between the desired lasing peak and the undesired sidebands in the lasing spectrum. **b**, **c** Measured (**b**) and simulated (**c**) *x*-polarized far-field emission patterns from the Dirac-vortex lasers with *R*_0_/*a*_0_ = 0.01, 0.5, 1, and 2. Increasing *R*_0_ leads to a decreased divergence angle. The Dirac-vortex laser with *R*_0_/*a*_0_ = 2 exhibits a half width at half maximum of the emission beam of 8.6°, as marked by the green dashed circles in **b** and **c**. **d**, **e** Measured (**d**) and simulated (**e**) *y*-polarized far-field emission patterns from the Dirac-vortex laser with *R*_0_/*a*_0_ = 0.01. The white double-headed arrows in **b**–**e** indicate the polarization direction for the corresponding far-field emission pattern. The white dashed circles in **b**–**e** indicate the numerical aperture (NA = 0.43) of the 50× collecting objective

The lasing properties of the Dirac-vortex cavity with different cavity sizes *R*_0_ were further investigated. Figure 4a shows the normalized experimental lasing spectra with different *R*_0_ values, confirming that the lasing wavelength is always at ~1340 nm. Theoretically, the lasing wavelength is topologically pinned to the Dirac point and thus does not vary with *R*_0_ (see Fig. S7a in the Supplementary Information). The fluctuation of the experimental lasing wavelength in Fig. 4a is attributed to the random variations of the structural parameter *s*_0_ introduced in device fabrication. The lasing spectra in Fig. 4a also suggest that the samples with a larger *R*_0_ exhibit a narrower lasing linewidth. This trend agrees well with the simulated *Q* factors of the MBS (see Fig. S7a in the Supplementary Information), indicating that the Dirac-vortex cavity with a larger *R*_0_ has a lower dissipation rate. Some sidebands emerge in the lasing spectrum of the Dirac-vortex cavity with *R*_0_/*a*_0_ = 2 and 4, as marked by the black arrows in Fig. 4a. The FSR as determined from the separation in wavelength between the MBS and its nearest sideband is 33.75 nm for *R*_0_/*a*_0_ = 2 and 25.14 nm for *R*_0_/*a*_0_ = 4. This suggests a reduction of FSR by 25.5% when *R*_0_/*a*_0_ is increased from 2 to 4. Our simulated results show that increasing *R*_0_/*a*_0_ from 2 to 4 leads to a nearly doubled modal area (see Fig. S7b in the Supplementary Information), which clearly suggests that the FSR of the Dirac-vortex cavities possesses an unconventional scaling law with the modal volume *V* that defies the FSR ∝ *V*^−1^ relation.

The far-field patterns and emission polarizations of the Dirac-vortex lasers with different cavity sizes *R*_0_ were also investigated. Figure 4b, c show the measured and simulated *x*-polarized far-field patterns from the Dirac-vortex lasers with *R*_0_/*a*_0_ = 0.01, 0.5, 1, and 2. The white dashed circles indicate the numerical aperture (NA = 0.43) of the collection objective. Figure 4d, e show the measured and simulated *y*-polarized far-field patterns from the Dirac-vortex laser with *R*_0_/*a*_0_ = 0.01, suggesting that the *y*-polarized electric field component is zero. The *y*-polarized electric field component from Dirac-vortex lasers with other cavity sizes is also near zero (see Fig. S8 in the Supplementary Information). The experimental and numerical results agree well with each other, confirming that the Dirac-vortex lasers have pure linear polarization along the *x* direction in their vertical emission in the far field. Besides, gradual increase in the pump intensity leads to improved directionality and polarization of the devices (see Fig. S9). Figure 4b, c also suggest that the emission directionality is gradually enhanced with an increased cavity size *R*_0_. A half width at half maximum of the emission beam of 8.6° (marked by the green dashed circles in Fig. 4b, c) can be obtained from the Dirac-vortex laser with *R*_0_/*a*_0_ = 2.

It is worth discussing the stability and reproducibility of the Dirac-vortex microcavity lasers. In principle, the III–V quantum dots are quite stable if properly encapsulated to avoid direct exposure to the ambient air. In our experiments, however, the quantum dots near the etched sidewalls were not encapsulated, which led to less stable devices due to photobleaching. This issue can be resolved by depositing a protecting layer to suppress the photo-bleaching effects. In addition, the cavity size of our devices was constrained by two factors: (i) the relatively weak in-plane optical confinement provided by the bandgap of the surrounding photonic crystal and (ii) the limited size of the entire photonic crystal slab due to the suspended-membrane configuration. By adopting the same concept of this work, an electrically pumped Dirac-vortex laser with a much larger cavity size and without the need for a suspended photonic crystal slab can readily be realized, which is expected to have improved lasing performance, such as narrower lasing linewidth, smaller far-field divergence, and higher output power. A possible experimental implementation would be following a configuration similar to the demonstrated electrically pumped photonic crystal surface-emitting lasers^35^ while replacing the in-plane photonic crystal pattern with the Dirac-vortex cavity in this work.

## Discussion

In conclusion, we experimentally demonstrated room-temperature continuous-wave Dirac-vortex topological lasers using InAs/InGaAs QD materials monolithically grown on a silicon substrate, obtaining single-mode linearly polarized vertical laser emission at a telecom wavelength. We confirmed that the lasing wavelength is topologically pinned to the Dirac point and that the FSR defies the universal FSR ∝ *V*^−1^ relation. These unique properties make our lasers fundamentally different from previous lasers with conventional cavity designs. These lasers are also the first topological lasers that are monolithically integrated on a silicon substrate and can operate under room-temperature continuous-wave conditions, marking an important step toward integrating topological lasers on the silicon nanophotonic and microelectronic platform. Considering that photonic crystal surface-emitting lasers have recently been commercialized, our Dirac-vortex lasers will find various practical applications including transmitters in fiber-based or free-space communication networks, light sources in Lidar or face-recognition systems, and chemical or biomedical sensors. Meanwhile, as lasers inherently exhibit non-Hermiticity^31^ and bosonic nonlinearity^32^, our results enable further experimental exploration of new physics in the MBS that have no counterpart in the electronic domain. By reducing the density of the InAs/InGaAs QDs such that each Dirac-vortex cavity contains only one QD, our devices provide an additional strategy for investigating the interplay between topology and quantum electrodynamics^33^.

During the revision of this manuscript, we became aware of related works on topological surface-emitting laser^36^ and quantum cascaded laser^37^.

## Materials and methods

### Numerical simulation

Commercial software (COMSOL Multiphysics) was used to calculate the bulk states and their eigenfrequencies of the photonic crystal. Commercial software (Lumerical) was used to conduct three-dimensional finite-difference time-domain simulation of the Dirac-vortex cavity.

### Epitaxial growth of InAs/InGaAs QDs on silicon

The Dirac-vortex topological lasers were fabricated in InAs/InGaAs QD layers monolithically grown on an on-axis silicon (001) substrate. High-temperature annealing (900 °C) with hydrogen atmosphere treatment was applied on a 300-mm-diameter silicon (001) substrate with a 0.15° misorientation in the [110] direction inside a metalorganic chemical vapor deposition system. A two-step 400-nm epitaxial GaAs film was grown to suppress the formation of antiphase boundaries. Then, the GaAs/silicon wafer was diced into 2-inch wafers for molecular beam epitaxy (MBE) growth. A 200-nm-thick GaAs buffer layer was deposited in the MBE chamber to achieve a smooth surface. Then, four sets of defect-filter layers were grown to reduce the density of threading dislocations owing to the large lattice mismatch between GaAs and silicon. Each defect-filter layer contains five repeats of In_0.18_Ga_0.82_As/GaAs strained-layer superlattice grown at 480 °C and a 300-nm GaAs spacer layer grown at 590 °C. The active layer was grown on a 1-µm-thick Al_0.6_Ga_0.4_As sacrificial layer. The active layer consists of four layers of InAs/In_0.15_Ga_0.85_As dot-in-well, which are separated by 50-nm GaAs spacer layers and sandwiched between 40-nm Al_0.4_Ga_0.6_As cladding layers. Each dot-in-well layer includes three monolayers of InAs deposited on a 2-nm In_0.15_Ga_0.85_As quantum well and capped by a 6-nm In_0.15_Ga_0.85_As layer.

### Device fabrication

Figure S3 provides the device fabrication process flowchart. First, 120-nm-thick SiO_2_ was deposited on the as-grown wafer by using plasma-enhanced chemical vapor deposition. Second, the patterns of the photonic crystal were defined in resist ZEP520A by electron-beam lithography. Third, the patterns were transferred to the SiO_2_ layer by using reactive-ion etching. Fourth, the residual resist was removed by using O_2_ plasma ashing. Fifth, the patterns of the photonic crystal were transferred from the SiO_2_ layer to the III–V materials by using chlorine-based inductively coupled plasma reactive-ion etching. Then, the residual SiO_2_ hard mask was removed by using diluted hydrofluoric acid. Finally, the 1-μm-thick Al_0.6_Ga_0.4_As sacrificial layer was removed by wet etching in a 40% hydrofluoric acid solution to form an air cladding underneath the photonic crystal slab.

### Device measurement

The fabricated devices were placed in a μ-PL measurement system with a surface-normal pump configuration at room temperature. A continuous-wave 632.8-nm He-Ne laser was used as the pump source. A 50× or 20× objective was used to focus the pump laser onto the center of the Dirac-vortex microcavity, whose position was precisely controlled by piezoelectric nanopositioners. The emission from the devices was collected by the same objective. Specifically, the 20× objective was used to obtain the results in Fig. 4a (*R*_0_/*a*_0_ = 4), and the 50× objective was used to obtain the results in Fig. 3a–e, Fig. 4a (*R*_0_/*a*_0_ = 0.01, 0.5, 1, and 2), and Fig. 4b–e. The calibrated pump beam radius is 3.04 μm for the 20× objective and 1.07 μm for the 50× objective. A long-pass filter was used to block the pump laser, and the emitted light was guided into a monochrometer with a thermoelectrically cooled InGaAs photodetector to characterize the emission spectra. A convex lens was used to project the emission from the device to a near-infrared camera, with the polarization state controlled by a linear polarizer, to characterize the far-field emission patterns.

### Supplementary information

Supplementary Information

## Data Availability

The data that support the findings of this study are available from the corresponding author upon reasonable request.
