# Supplementary material for: Room-temperature continuous-wave topological Dirac-vortex microcavity lasers on silicon
Source: Light Sci Appl. 2023 Oct 24;12:255. doi: 10.1038/s41377-023-01290-4 (PMC10593858; doi:10.1038/s41377-023-01290-4)
Supplement: Supplementary file 1 — Supplementary Information [file 41377_2023_1290_MOESM1_ESM.pdf]

Supplementary Information for "Room-temperature continuous-wave topological Dirac-vortex microcavity lasers on silicon"

Jingwen Ma[1,2,†], Taojie Zhou[2,3,†], Mingchu Tang[3,†], Haochuan Li[2], Zhan Zhang[2], Xiang Xi[1], Mickael Martin[4], Thierry Baron[4], Huiyun Liu[3], Zhaoyu Zhang[2,*], Siming Chen[3,*] and Xiankai Sun[1,*]

[1]*Department of Electronic Engineering, The Chinese University of Hong Kong, Shatin, New Territories, Hong Kong SAR, China*

[2]*School of Science and Engineering, The Chinese University of Hong Kong, Shenzhen, Guangdong 518172, China*

[3]*Department of Electronic and Electrical Engineering, University College London, London, WC1E 7JE, United Kingdom*

[4]*Université Grenoble Alpes, CNRS, CEA-LETI, MINATEC, Grenoble INP, LTM, F-38054 Grenoble, France*

[†]*These authors contributed equally to this work*

[*]*Corresponding author: zhangzy@cuhk.edu.cn (Z.Z.); siming.chen@ucl.ac.uk (S.C.); xksun@cuhk.edu.hk (X.S.)*

Contents

1. Theoretical analysis 2

1.1. Effective bulk Hamiltonian 2

1.2. Analytical solution of the Dirac-vortex states 4

1.3. Comparison with the Kekulé distortion scheme 7

2. Additional methods and results 9

1. Theoretical analysis

1.1. Effective bulk Hamiltonian

Due to the symmetric spatial distribution of refractive index in the z direction, the TM and TE modes supported by the photonic crystal slab are orthogonal to each other. We focus on the TE modes which exhibit nonzero magnetic components only in the z direction. The Maxwell equation for the magnetic field $h_z(\mathbf{r})$ with harmonic time dependence is

$$\nabla\times\left[\frac{1}{\varepsilon(\mathbf{r})}\nabla\times h_z(\mathbf{r})\right]=\frac{\omega^2}{c^2}h_z(\mathbf{r}) \tag{S1}$$

A Bloch mode with momentum $\mathbf{k}$ can be expanded in an orthonormal set of plane waves as

$$h_z(\mathbf{r})=\exp\left(j\mathbf{k}\cdot\mathbf{r}\right)\sum_{\mathbf{G}}c_{\mathbf{k},\mathbf{G}}\exp(j\mathbf{G}\cdot\mathbf{r}) \tag{S2}$$

where $\mathbf{G}$ is a reciprocal vector of the photonic crystal. Here, we take six reciprocal vectors $\mathbf{G}_m$ (m = 1–6) into consideration and obtain an eigenvalue problem

$$\begin{aligned}\frac{\omega_{\mathbf{k}}^2}{c^2}c_{\mathbf{k},\mathbf{G}'}&=\sum_{\mathbf{G}}c_{\mathbf{k},\mathbf{G}}\cdot\eta(\mathbf{G}-\mathbf{G}')\cdot\left[\left(\mathbf{k}+\mathbf{G}\right)\cdot\left(\mathbf{k}+\mathbf{G}'\right)\right]\\&\approx\sum_{\mathbf{G}}c_{\mathbf{k},\mathbf{G}}\cdot\eta(\mathbf{G}-\mathbf{G}')\cdot\left[\mathbf{G}\cdot\mathbf{G}'+\mathbf{k}\cdot\left(\mathbf{G}+\mathbf{G}'\right)\right]\end{aligned} \tag{S3}$$

where $\eta(\mathbf{k})$ is the Fourier transform of $\varepsilon^{-1}(\mathbf{r})$:

$$\eta(\mathbf{k})=\int_{\text{unit cell}}\varepsilon^{-1}(\mathbf{r})\exp(j\mathbf{k}\cdot\mathbf{r})\cdot d\mathbf{r}$$

We will find out the values of $\eta(\mathbf{k})$ at the $\mathbf{\Gamma}$, $\mathbf{G}_m$, and $\mathbf{P}_m$ points (m = 1–6), which are shown in Fig. S1. Specifically, the geometric parameter δ_t leads to a nonzero $\eta(\mathbf{G}_m)=\alpha_t$ (m = 1–6), and the other geometric parameter δ_i leads to a nonzero imaginary part of $\eta(\mathbf{P}_m)$ such that $\eta(\mathbf{P}_m)=\eta_1+j\cdot\alpha_i$ for m = 1–3 and $\eta(\mathbf{P}_m)=\eta_1-j\cdot\alpha_i$ for m = 4–6. Here, α_t and α_i are proportional to the geometric parameter δ_t and δ_i, respectively. Then we have

$$\lambda_{\mathbf{k}}\mathbf{c}_{\mathbf{k}}=\mathbf{H}_{\mathbf{k}}\mathbf{c}_{\mathbf{k}} \tag{S4}$$

with the eigenstate vector $\mathbf{c_k} = (c_{\mathbf{k},\mathbf{G}_{1,+}}, c_{\mathbf{k},\mathbf{G}_{2,+}}, c_{\mathbf{k},\mathbf{G}_{3,+}}, c_{\mathbf{k},\mathbf{G}_{1,-}}, c_{\mathbf{k},\mathbf{G}_{2,-}}, c_{\mathbf{k},\mathbf{G}_{3,-}})^{\mathrm{T}}$, the eigenvalue $\lambda_\mathbf{k} = \omega_\mathbf{k}^2/c^2 - G^2(\eta_0 + \eta_1/2)$, and the Hamiltonian

$$\mathbf{H_k} = (\eta_0 \cdot \mathbf{I}_3 \otimes \boldsymbol{\sigma}_0 - \eta_1/2 \cdot \mathbf{M}_1 \otimes \boldsymbol{\sigma}_0 + \alpha_t \cdot \mathbf{M}_1 \otimes \boldsymbol{\sigma}_x + j\alpha_i \cdot \mathbf{M}_2 \otimes \boldsymbol{\sigma}_z) \cdot \mathbf{M_k} - G^2(\eta_0 + \eta_1/2) \cdot \mathbf{I}_3 \otimes \boldsymbol{\sigma}_0.$$

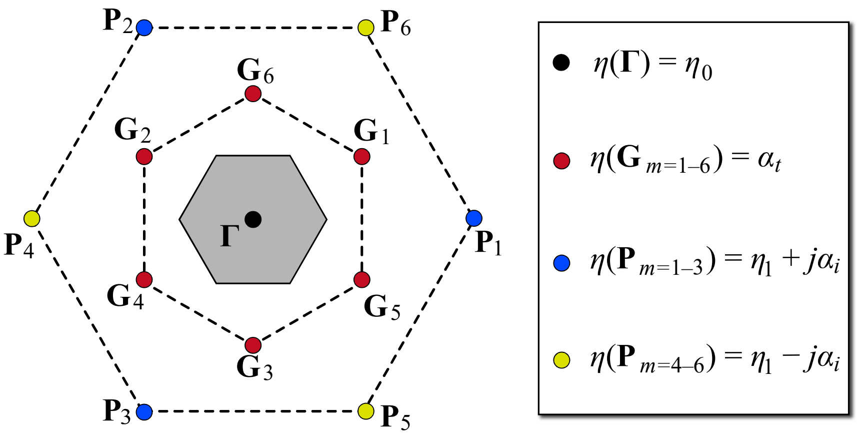

Figure S1. Theoretical analysis of the photonic crystal using a plane-wave expansion method. The gray shaded hexagon indicates the first Brillouin zone of the photonic crystal. The distribution of $\eta(\mathbf{k})$ is similar to that in our previous work on nanomechanical systems[30]. Adapted with permission from Ref. 30.

In the above equations, the symbols are defined as $G = |\mathbf{G}_m|$ (m = 1–3),

$$\boldsymbol{\sigma}_0 = \begin{bmatrix} 1 & 0 \\ 0 & 1 \end{bmatrix}, \boldsymbol{\sigma}_x = \begin{bmatrix} 0 & 1 \\ 1 & 0 \end{bmatrix}, \boldsymbol{\sigma}_z = \begin{bmatrix} 1 & 0 \\ 0 & -1 \end{bmatrix},$$

$$\mathbf{I}_3 = \begin{bmatrix} 1 & 0 & 0 \\ 0 & 1 & 0 \\ 0 & 0 & 1 \end{bmatrix}, \mathbf{M}_1 = \begin{bmatrix} 0 & 1 & 1 \\ 1 & 0 & 1 \\ 1 & 1 & 0 \end{bmatrix}, \mathbf{M}_2 = \begin{bmatrix} 0 & 1 & -1 \\ -1 & 0 & 1 \\ 1 & -1 & 0 \end{bmatrix}, (\mathbf{M_k})_{i,j} = \mathbf{G}_i \cdot \mathbf{G}_j + \mathbf{k} \cdot (\mathbf{G}_i + \mathbf{G}_j).$$

At the $\boldsymbol{\Gamma}$ point ($\mathbf{k} = 0$), the Hamiltonian $\mathbf{H_k}$ has eigenvalues

$$(\lambda_{1,\uparrow}, \lambda_{2,\uparrow}, \lambda_{1,\downarrow}, \lambda_{2,\downarrow}) = (-\Delta_0/2, \Delta_0/2, -\Delta_0/2, \Delta_0/2)$$

As the opened bandgap $\Delta_0 = G^2\sqrt{3\alpha_i^2 + \alpha_t^2}$ is proportional to the geometric parameter δ_0 and does not depend on θ, the parameters α_i and α_t can be expressed as $(\sqrt{3}\alpha_i, \alpha_t) = \Delta_0/G^2 \cdot (\cos\theta, \sin\theta)$. Note that the Hamiltonian in Eq. (S4) has six eigenfrequencies in total, but we only need to focus on four of them, because the other two have eigenvalues far away from $\lambda_{n,\uparrow\downarrow}$ (n = 1, 2). We define another four states which are superpositions of the plane waves with wave vectors $\mathbf{G}_m$ (m = 1–6)

$$\begin{cases} \left|\psi_{\pm,\downarrow}\right\rangle = \frac{1}{\sqrt{6}}\sum_{m=1}^{3} e^{-j2\pi m/3}\cdot\left(e^{-j\mathbf{G}_m\cdot\mathbf{r}} \pm je^{j\mathbf{G}_m\cdot\mathbf{r}}\right) \\ \left|\psi_{\pm,\uparrow}\right\rangle = \frac{1}{\sqrt{6}}\sum_{m=1}^{3} e^{j2\pi m/3}\cdot\left(\pm e^{j\mathbf{G}_m\cdot\mathbf{r}} + je^{-j\mathbf{G}_m\cdot\mathbf{r}}\right) \end{cases} \tag{S5}$$

so that $h_z(\mathbf{r})$ can be decomposed as

$$h_z(\mathbf{r}) = \exp(j\mathbf{k}\cdot\mathbf{r})\cdot\sum_{n,s} c_{\mathbf{k},n,s}\left|\psi_{n,s}\right\rangle, \quad (n=\pm, s=\downarrow\uparrow) \tag{S6}$$

With the states $\left(\left|\psi_{+,\downarrow}\right\rangle, \left|\psi_{+,\uparrow}\right\rangle, \left|\psi_{-,\downarrow}\right\rangle, \left|\psi_{-,\uparrow}\right\rangle\right)$ as the basis, Eq. (S4) can be reduced to

$$\lambda_{\mathbf{k}}\mathbf{c}_{\mathbf{k}} = \mathbf{H}(\mathbf{k})\cdot\mathbf{c}_{\mathbf{k}} \tag{S7}$$

with the eigenstate vector $\mathbf{c}_{\mathbf{k}} = (c_{\mathbf{k},+,\downarrow}, c_{\mathbf{k},+,\uparrow}, c_{\mathbf{k},-,\downarrow}, c_{\mathbf{k},-,\uparrow})^{\mathrm{T}}$ and the Hamiltonian

$$\mathbf{H}(\mathbf{k}) = v_D\cdot(\boldsymbol{\sigma}_x k_x + \boldsymbol{\sigma}_y k_y) + \frac{\Delta_0}{2}\boldsymbol{\sigma}_z(\boldsymbol{\tau}_x\cos\theta - \boldsymbol{\tau}_y\sin\theta) \tag{S8}$$

where $\boldsymbol{\sigma}_x$, $\boldsymbol{\sigma}_y$, $\boldsymbol{\sigma}_z$, and $\boldsymbol{\tau}_z$ are the Pauli matrices, and $v_D = G\cdot(\eta_1 - \eta_0)$ is the effective Fermi velocity near the $\boldsymbol{\Gamma}$ point. In Eq. (S8), the first term indicates the double-Dirac-cone dispersion relation in the momentum space, and the second term indicates the effective masses that produce the bulk bandgap. The Hamiltonian $\mathbf{H}(\mathbf{k})$ in Eq. (S8) is mathematically identical to the Jackiw–Rossi model, where $\left|\psi_{\pm,\downarrow\uparrow}\right\rangle$ represents charge-conjugate (+/−) Dirac fermions with opposite spins (↓/↑). Besides, similar to the Jackiw–Rossi model, the states $\left|\psi_{\pm,\downarrow\uparrow}\right\rangle$ in Eq. (S5) naturally satisfy the charge-conjugation symmetry

$$\begin{pmatrix} \left|\psi_{-,\downarrow}\right\rangle \\ \left|\psi_{-,\uparrow}\right\rangle \end{pmatrix} = -j\begin{pmatrix} \left|\psi_{+,\downarrow}\right\rangle \\ \left|\psi_{+,\uparrow}\right\rangle \end{pmatrix}^{\dagger}\boldsymbol{\sigma}_y \tag{S9}$$

1.2. Analytical solution of the Dirac-vortex states

Our previous discussion focuses only on bulk states in strictly periodic photonic crystal structures with constant parameters Δ_0 and θ. Next, we will focus on a different case where the geometric

parameters Δ_0 and θ are functions of the spatial position $\mathbf{r}$. Similar to Eqs. (S7) and (S8), the Dirac-vortex state is governed by

$$\lambda_0 \mathbf{c}(\mathbf{r}) = \mathbf{H}(\mathbf{r}) \cdot \mathbf{c}(\mathbf{r}) \tag{S10}$$

with the spatially dependent vector $\mathbf{c}(\mathbf{r}) = [c_{+,\downarrow}(\mathbf{r}), c_{+,\uparrow}(\mathbf{r}), c_{-,\downarrow}(\mathbf{r}), c_{-,\uparrow}(\mathbf{r})]^{\mathrm{T}}$, the eigenvalue $\lambda_0 = \omega_0^2/c^2 - G^2(\eta_0 + \eta_1/2)$, and the real-space Hamiltonian

$$\mathbf{H}(\mathbf{r}) = -jv_D \cdot (\boldsymbol{\sigma}_x \partial_x + \boldsymbol{\sigma}_y \partial_y) + \frac{\Delta_0}{2} \boldsymbol{\sigma}_z (\boldsymbol{\tau}_x \cos\theta - \boldsymbol{\tau}_y \sin\theta) \tag{S11}$$

In the polar coordinate system, $\mathbf{r} = R \cdot (\cos\varphi, \sin\varphi)$. We focus on the zero mode with $\omega_0 = cG\sqrt{\eta_0 + \eta_1/2}$ (i.e., $\lambda_0 = 0$), so that Eqs. (S10) and (S11) lead to the following equations

$$\begin{cases} -jv_{\mathrm{D}} e^{-j\varphi} \left(\partial_R - \dfrac{j}{R} \partial_\varphi \right) c_{+,\uparrow}(\mathbf{r}) + \dfrac{\Delta_0(\mathbf{r}) e^{j\theta(\mathbf{r})}}{2} c_{+,\uparrow}^*(\mathbf{r}) = 0 \\ -jv_{\mathrm{D}} e^{j\varphi} \left(\partial_R + \dfrac{j}{R} \partial_\varphi \right) c_{+,\downarrow}(\mathbf{r}) + \dfrac{\Delta_0(\mathbf{r}) e^{j\theta(\mathbf{r})}}{2} c_{+,\downarrow}^*(\mathbf{r}) = 0 \end{cases} \tag{S12}$$

Note that the values of $c_{-,\downarrow\uparrow}(\mathbf{r})$ can be determined by the relationship $c_{-,\downarrow}(\mathbf{r}) = c_{+,\uparrow}^*(\mathbf{r})$ and $c_{-,\uparrow}(\mathbf{r}) = -c_{+,\downarrow}^*(\mathbf{r})$. We focus on a special case of $\Delta_0(R) = \Delta_{\max} \cdot [\tanh(R/R_0)]^4$ and $\theta(\varphi) = w \cdot \varphi + \theta_0$, where R_0 controls the size of the cavity, $w = 1$ is the winding number of the vortex, and θ_0 is the value of $\theta(\varphi)$ at $\varphi = 0$. We assume that the solution of Eq. (S12) is $c_{+,\downarrow\uparrow}(\mathbf{r}) = g_{\downarrow\uparrow}(R) \cdot \exp\left(jp_{\downarrow\uparrow}\varphi + j\vartheta_{\downarrow\uparrow}\right)$, where $g_{\downarrow\uparrow}(R)$ is the amplitude distribution along the radial direction, the integer $p_{\downarrow\uparrow}$ is the angular quantum number, and $\vartheta_{\downarrow\uparrow}$ is the additional phase term of $c_{+,\downarrow\uparrow}(\mathbf{r})$. Equation (S12) can be rewritten as

$$\begin{cases} -jv_{\mathrm{D}} \left(\partial_R + \dfrac{p_\uparrow}{R} \right) g_\uparrow(R) + \dfrac{\Delta_0(R)}{2} g_\uparrow(R) e^{-2j\left(p_\uparrow \varphi + \vartheta_\uparrow - \theta_0/2\right)} = 0 \\ -jv_{\mathrm{D}} \left(\partial_R - \dfrac{p_\downarrow}{R} \right) g_\downarrow(R) + \dfrac{\Delta_0(R)}{2} g_\downarrow(R) e^{-2j\left(p_\downarrow \varphi + \varphi + \vartheta_\downarrow - \theta_0/2\right)} = 0 \end{cases} \tag{S13}$$

As Eq. (S13) is valid for arbitrary values of φ, we obtain $p_\uparrow = 0$, and $p_\downarrow = -1$, so that

$$\begin{cases} \partial_R g_\uparrow(R) = \dfrac{\Delta_0(R)}{2v_\mathrm{D}} g_\uparrow(R) e^{-2j(\vartheta_\uparrow - \theta_0/2 + \pi/4)} \\ \partial_R g_\downarrow(R) = \dfrac{1}{R} g_\downarrow(R) + \dfrac{\Delta_0(R)}{2v_\mathrm{D}} g_\downarrow(R) e^{-2j(\vartheta_\downarrow - \theta_0/2 + \pi/4)} \end{cases} \tag{S14}$$

Besides, as $g_{\downarrow\uparrow}(R)$ is always real, the phase terms in Eq. (S14) have to satisfy $\vartheta_{\uparrow\downarrow} = -\theta_0/2 + \pi/4$ or $\vartheta_{\uparrow\downarrow} = -\theta_0/2 - \pi/4$, so that Eq. (S13) can be reduced to

$$\begin{cases} \partial_R g_\uparrow(R) = \dfrac{\Delta_0(R)}{2v_\mathrm{D}} g_\uparrow(R) \quad \left(\vartheta_\uparrow = -\theta_0/2 + \dfrac{\pi}{4}\right) \\ \partial_R g_\uparrow(R) = -\dfrac{\Delta_0(R)}{2v_\mathrm{D}} g_\uparrow(R) \quad \left(\vartheta_\uparrow = -\theta_0/2 - \dfrac{\pi}{4}\right) \end{cases} \tag{S15}$$

and

$$\begin{cases} \partial_R g_\downarrow(R) = \left(-\dfrac{1}{R} + \dfrac{\Delta_0(R)}{2v_\mathrm{D}}\right) g_\downarrow(R) \quad \left(\vartheta_\downarrow = -\theta_0/2 + \dfrac{\pi}{4}\right) \\ \partial_R g_\downarrow(R) = \left(-\dfrac{1}{R} - \dfrac{\Delta_0(R)}{2v_\mathrm{D}}\right) g_\downarrow(R) \quad \left(\vartheta_\downarrow = -\theta_0/2 - \dfrac{\pi}{4}\right) \end{cases} \tag{S16}$$

Considering the boundary conditions that $g_{\downarrow\uparrow}(R = 0)$ is finite and $g_{\downarrow\uparrow}(R = +\infty)$ is zero, we find that Eq. (S15) has a nonzero solution

$$g_\uparrow(R) = \exp\left[-\int_0^R \frac{\Delta_0(R)}{2v_\mathrm{D}} dr\right] \tag{S17}$$

only when $\vartheta_\uparrow = -\theta_0/2 - \pi/4$, while Eq. (S16) always has a zero solution $g_\downarrow(R) = 0$.

In conclusion, the modal profile of the Dirac-vortex state with parameters $\Delta_0(R) = \Delta_{\max}\cdot[\tanh(R/R_0)]^4$ and $\theta(\varphi) = \varphi + \theta_0$ is

$$h_z(\mathbf{r}) = g_0(R)\cdot|\psi_0\rangle \tag{S18}$$

where the envelope function $g_0(R)$ controlling the modal volume of the Dirac-vortex state is

$$g_0(R)=\exp\left[\frac{\Delta_{\max}R_0}{6v_D}\cdot\left(\tanh^3\frac{R}{R_0}+3\tanh\frac{R}{R_0}\right)\right]\cdot\exp\left(-\frac{\Delta_{\max}R}{2v_D}\right) \tag{S19}$$

and the Bloch mode $|\psi_0\rangle = e^{-j(\pi/4+\theta_0/2)}|\psi_{+,\uparrow}\rangle + e^{j(\pi/4+\theta_0/2)}|\psi_{-,\downarrow}\rangle$ controlling the detailed modal profile is

$$|\psi_0\rangle = e^{-j(\pi/4+\theta_0/2)}|\psi_{+,\uparrow}\rangle + e^{j(\pi/4+\theta_0/2)}|\psi_{-,\downarrow}\rangle \tag{S20}$$

Note that $h_z(\mathbf{r})$ naturally satisfies $h_z(\mathbf{r}) = h_z^*(\mathbf{r})$. From Eq. (S20) one can also find that the Dirac-vortex mode exhibits an interesting property: adiabatically varying θ_0 from 0 to 2π introduces a nontrivial geometric phase π. This phenomenon is closely related to the braiding of the Majorana modes.

1.3. Comparison with the Kekulé distortion scheme

To investigate the relationship and difference between our scheme and the Kekulé distortion scheme widely used by others, we begin with the original uniform structure without any geometric variations. In this case, the physics is generally described by a four-band Dirac Hamiltonian

$$\mathbf{H} = k_x\mathbf{\Gamma}_1 + k_y\mathbf{\Gamma}_2$$

where $\mathbf{\Gamma}_i$ (i = 1–5) is the 4 × 4 Gamma matrix satisfying the anticommutation relation $\{\mathbf{\Gamma}_i, \mathbf{\Gamma}_j\} = 2\delta_{ij}$ similar to the 2 × 2 Pauli matrix in two-band Dirac Hamiltonian. To ensure the four-fold degeneracy at the Dirac point, the four-band Dirac Hamiltonian is composed by at most five Gamma matrices, which means that there are at most three synthetic parameters δ_i, δ_t, δ_{t2} to make a full Hamiltonian

$$\mathbf{H} = k_x\mathbf{\Gamma}_1 + k_y\mathbf{\Gamma}_2 + \delta_i\mathbf{\Gamma}_3 + \delta_t\mathbf{\Gamma}_4 + \delta_{t2}\mathbf{\Gamma}_5$$

In our specific case, the detailed form of Gamma matrix is $(\mathbf{\Gamma}_1, \mathbf{\Gamma}_2, \mathbf{\Gamma}_3, \mathbf{\Gamma}_4, \mathbf{\Gamma}_5) = (\boldsymbol{\sigma}_x, \boldsymbol{\sigma}_y, \boldsymbol{\sigma}_z\boldsymbol{\tau}_x, -\boldsymbol{\sigma}_z\boldsymbol{\tau}_y, \boldsymbol{\tau}_z\boldsymbol{\sigma}_z)$. As shown in Fig. S2, these three parameters can span a three-dimensional (3D) synthetic parameter space. Our scheme operates in the 2D subspace spanned by δ_i and δ_t, while the

Kekulé scheme operates in the 2D subspace spanned by δ_t and δ_{t2}. In fact, any big circles of the sphere shown in Fig. S2 can lead to a Dirac-vortex cavity. Different choices of the big circles can lead to different near-field modal profiles as well as far-field patterns. This interesting phenomenon was investigated and demonstrated on a nanomechanical platform (see Fig. 5 in Ref. 30).

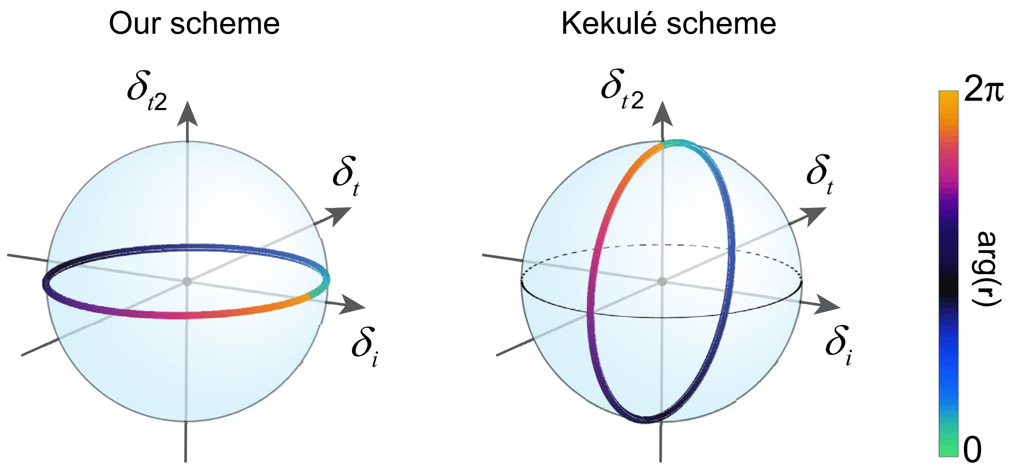

Figure S2. Illustration of the extended 3D synthetic parameter space. Our scheme and the Kekulé scheme are both based on a mapping from the azimuthal angle of spatial domain arg(**r**) to the 3D synthetic parameter space. Our scheme operates in the 2D subspace spanned by parameters δ_i and δ_t, while the Kekulé scheme operates in the 2D subspace spanned by δ_t and δ_{t2}. The difference between our scheme and the Kekulé scheme has been discussed in detail in our previous work on nanomechanical systems[30]. Adapted with permission from Ref. 30.

2. Additional methods and results

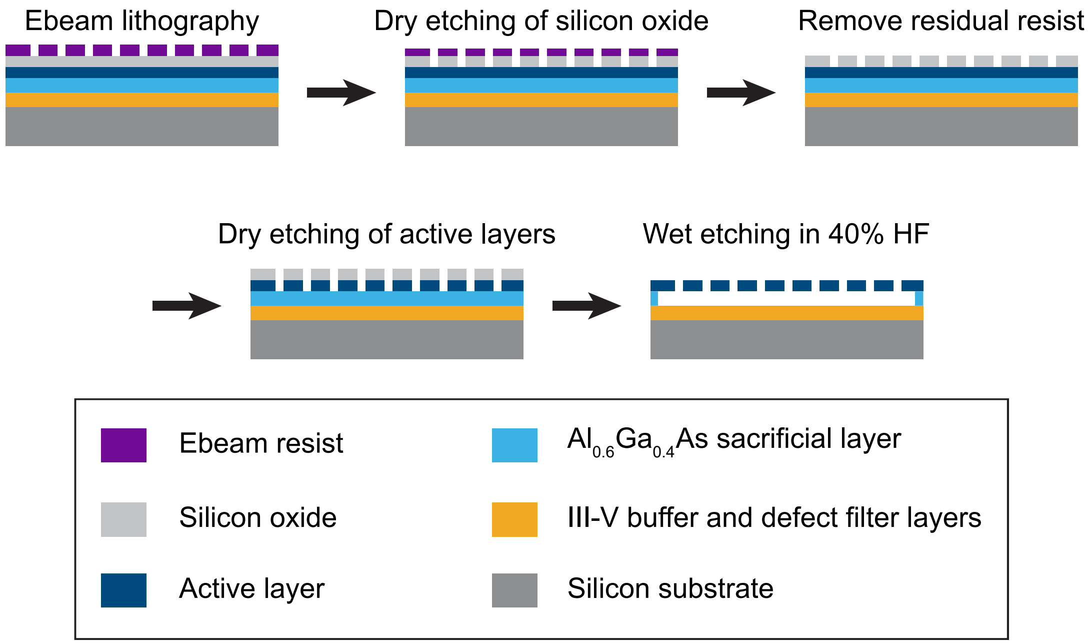

Figure S3. Device fabrication process flowchart.

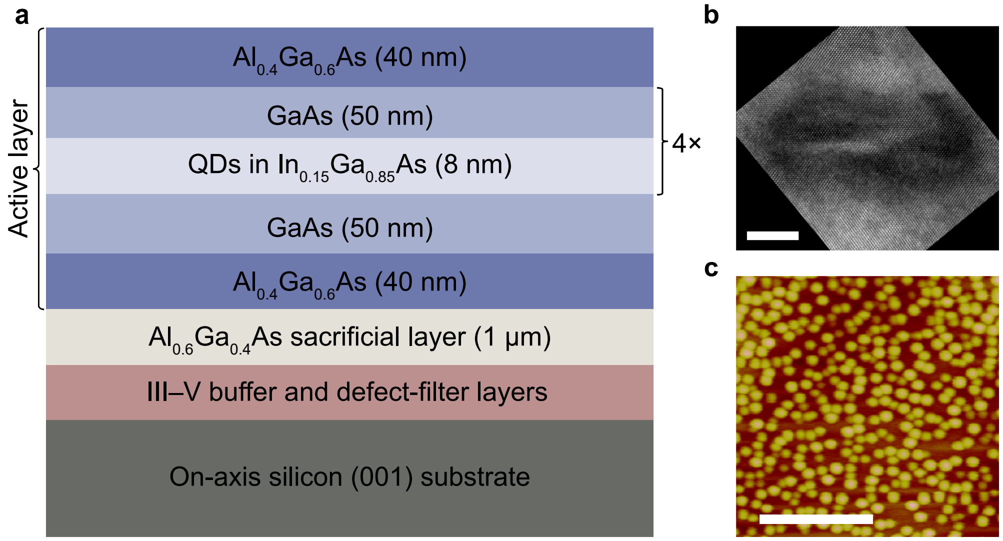

Figure S4. Epitaxial structure of the InAs/InGaAs QD active layer grown on silicon. a, Illustration of epitaxial structure of the active layer, which consists of two symmetric 40-nm-thick $Al_{0.4}Ga_{0.6}As$ cladding layers and four layers of InAs/InGaAs dot-in-well structures separated by 50-nm GaAs spacer layers. **b**, Cross-sectional bright-field transmission electron microscope image of a single QD. Scale bar, 10 nm. **c**, Atomic force microscope image of uncapped InAs/InGaAs QDs. Scale bar, 400 nm. The same wafer was also used for fabricating the corner-state lasers[34]. Adapted with permission from Ref. 34. © 2022 American Chemical Society.

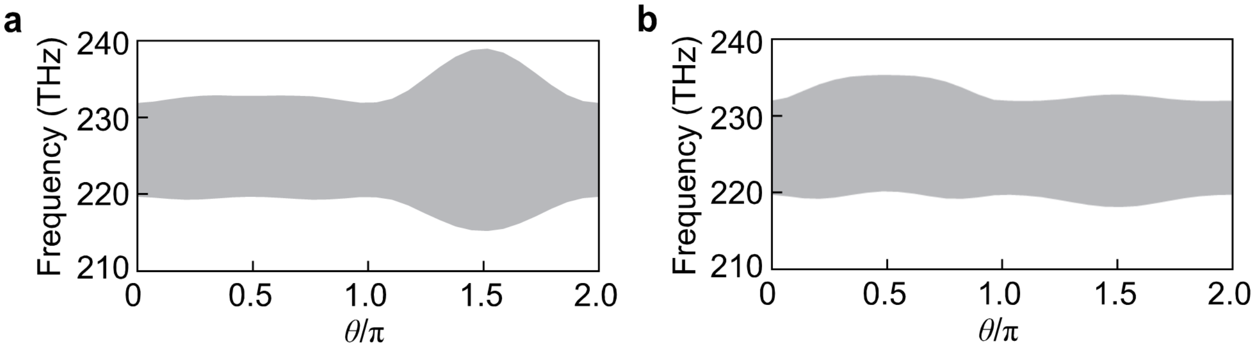

Figure S5. Dependence of the bulk bandgap on parameter *θ*. a, **b**, Simulated bulk bandgaps at the Γ point of the first Brillouin zone with δ_0 fixed at 35 nm and θ varying from 0 to 2π. The geometric parameters δ_t and δ_i are determined by $(\delta_t, \delta_i) = \delta_0(\alpha \cdot \sin\theta, \cos\theta)$. The simulated bulk bandgap depends strongly on θ when α is 0.49 (**a**). In our experiment, we set $\alpha = 0.65$ (0.33) for $\delta_t > 0$ ($\delta_t < 0$) to obtain a weakly θ-dependent bulk bandgap (**b**).

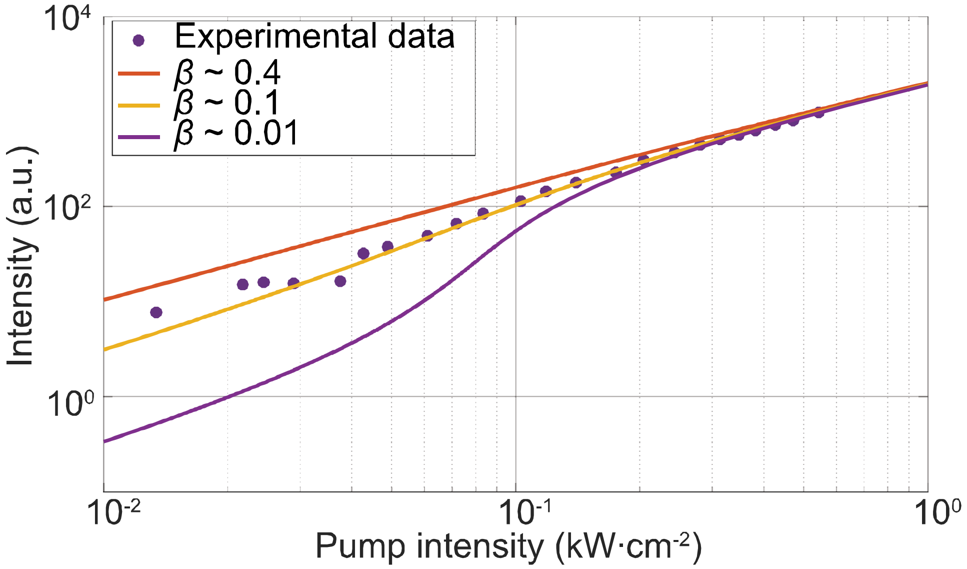

Figure S6. Log–log plot of the *L*–*L* curve for the sample shown in Fig. 3b and the theoretically calculated curves by using the coupled rate equations with different *β* values.

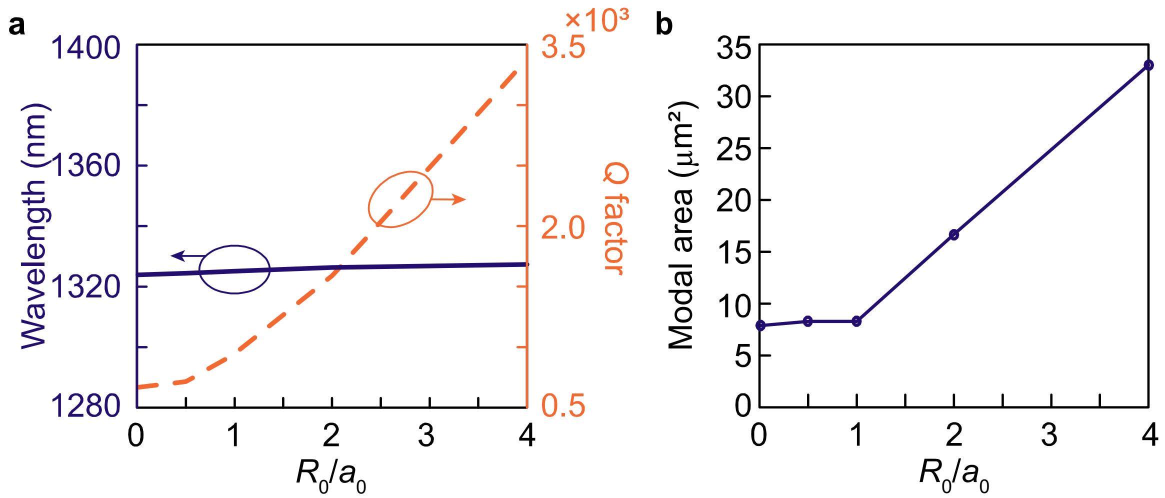

Figure S7. Simulated properties of the Dirac-vortex laser cavities with different *R_0*. a, Simulated cavity resonant wavelength (purple solid line) and *Q* factor (orange dashed line) of the Dirac-vortex lasers as a function of the cavity size R_0. **b**, Simulated modal area of the Majorana bound state as a function of the cavity size R_0. The modal area increases from 16.7 to 33.0 μm^2 when R_0/a_0 is increased from 2 to 4.

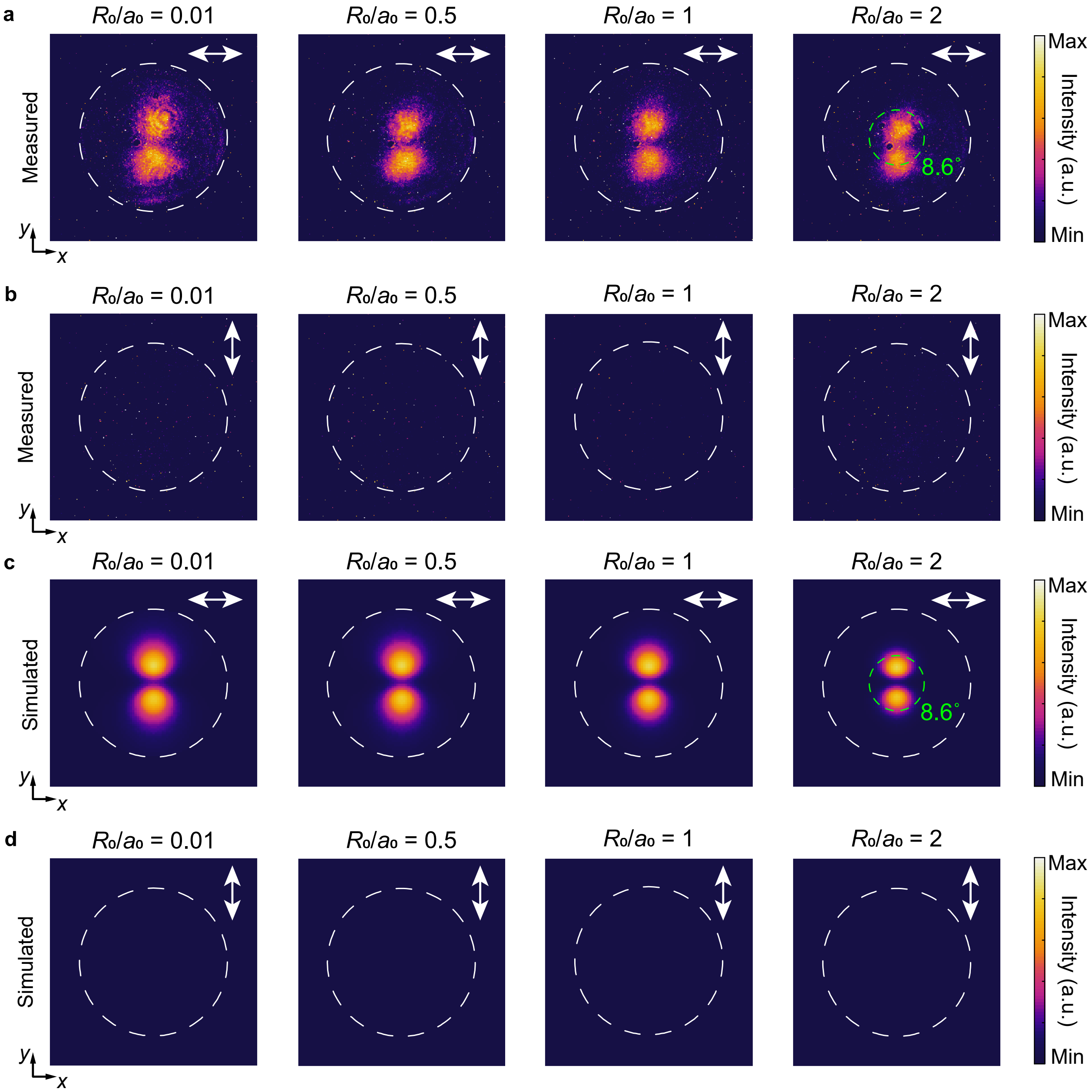

Figure S8. Measured and simulated far-field patterns from devices with different cavity sizes and polarization directions.

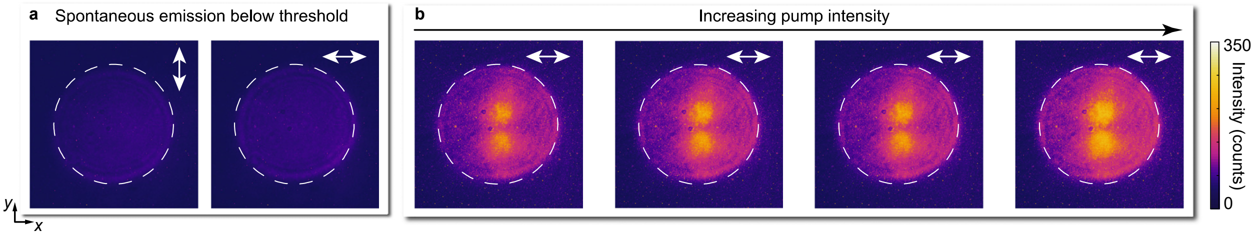

Figure S9. Measured far-field patterns of the device with R_0/a_0 = 1 under varying pump intensities. **a**, Measured spontaneous emission below the lasing threshold. The spontaneous emission does not exhibit directionality or polarization. **b**, Measured far-field patterns above the lasing threshold. The peanut-shaped lasing pattern appears gradually in the *x*-polarized emission as the pump intensity increases, which suggests that the directionality and polarization of the device are improved with increased pump intensity.

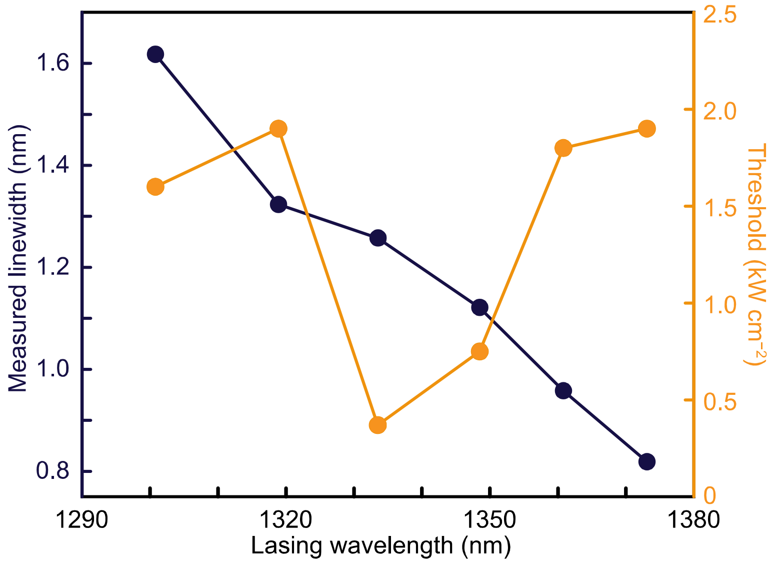

Figure S10. Measured lasing wavelength, linewidth, and threshold of devices with different s_0. Varying the size of etched holes s_0 leads to effective tuning of the lasing wavelength from 1300 to 1370 nm. Under a pump intensity of 4.25 kW cm^{-2}, the devices with a smaller s_0 exhibit a narrower linewidth and a longer lasing wavelength. The narrower linewidth is attributed to a higher optical Q factor of the Dirac-vortex cavity with smaller etched holes which cause less optical scattering into the free space. The lasing threshold varies between 0.4 and 1.9 kW cm^{-2} and does not change monotonically with s_0. This is because the lasing threshold depends not only on the optical Q factor of the cavity but also on the gain coefficient of the material. Although the optical Q factor of the cavity increases with wavelength, the gain coefficient of the quantum dots drops at wavelengths longer than 1360 nm.
